# HPLC Method for Simultaneous Determination of Impurities and Degradation Products in Zonisamide

**DOI:** 10.4103/0250-474X.58183

**Published:** 2009

**Authors:** E. K. S. Vijayakumar, D. M. Dhore, M. Kumar

**Affiliations:** Mylan India Pvt. Ltd., Plot 1A/2, M. I. D. C. Industrial Estate, Taloja, Panvel-410 208, India; 1 Astrix Laboratories Ltd., Gaddapotharam, Kazipally, Hyderabad-502319, India

**Keywords:** Zonisamide, HPLC, method development, validation, related substances

## Abstract

A gradient reversed phase HPLC method was developed and validated for the analysis of related substances in zonisamide (1,2-benzisoxazole-3-methanesulfonamide), using a Waters Symmetry C8 (150*3.9 mm) column with a flow rate of 1.0 ml/min and detection at 280 nm. The mobile phase component A consisted of a mixture of 0.02 M aqueous potassium dihydrogen phosphate-acetonitrile-methanol (75:10:15 v/v/v), pH adjusted to 4.0 with orthophosphoric acid. The mobile phase component B consisted of a mixture of 0.02 M aqueous potassium dihydrogen phosphate-acetonitrile-methanol (15:40:45 v/v/v), pH 2.0 with orthophosphoric acid. The limit of detection and limit of quantitation were in the range of 0.001-0.007% and 0.0035-0.25% respectively with respect to sample concentration of 2 mg/ml. The method was linear in the range of LOQ level to 200% of specified limits for II-VIII (< 0.10%, r^2^= 0.9958-0.9999). The method is sensitive, specific, linear, accurate, precise and stability-indicating for the detection and quantitation of precursors (viz., 4-hydroxycoumarin, 1,2-benzisoxazole-3-acetic acid, 1,2-benzisoxazole-3-bromoacetic acid, 1,2-benzisoxazole-3-methylbromide, sodium 1,2-benzisoxazole-3-methanesulfonate), process impurities (viz., 2-hydroxyacetophenone oxime and 3,3,3-tribromomethyl-1,2-benzisoxazole) and drug degradation products formed under stress conditions.

Zonisamide (I), chemically known as 1,2-benzisoxazole-3-methanesulfonamide and belonging to the class of sulfonamides, is used for the treatment of epilepsy[1–3]. During the synthesis of I by the route reported in literature[4], the starting material 4-hydroxycoumarin (II), four precursors, *viz*., 1,2-benzisoxazole-3-acetic acid (III), 1,2-benzisoxazole-3-bromoacetic acid (IV), 1,2-benzisoxazole-3-methylbromide (V), sodium 1,2-benzisoxazole-3-methylbromide (VI) and two process impurities, *viz*., 2-hydroxyacetophenoneoxime (VII) and 3,3,3-tribromomethyl-1,2-benzisoxazole (VIII) shown in fig. 1 were considered as potential impurities. The content of all these, which could be present at trace levels, was needed to be analyzed in the drug substance. Among the high performance capillary electrophoresis (HPCE) and high performance liquid chromatography (HPLC) methods reported in literature[5–12] for the determination of I, none was found to be suitable for the simultaneous detection and quantification of II-VIII. This was due to poor resolution between I and VI. This necessitated development of a HPLC method suitable for the separation and simultaneous analysis of I and impurities II-VIII. The method was validated according to ICH Q1A (R2), Q2A and Q2B guidelines[13–15] and also extended to separation of degradation products formed under various stress conditions. The results are reported in this paper.

**Fig. 1:**
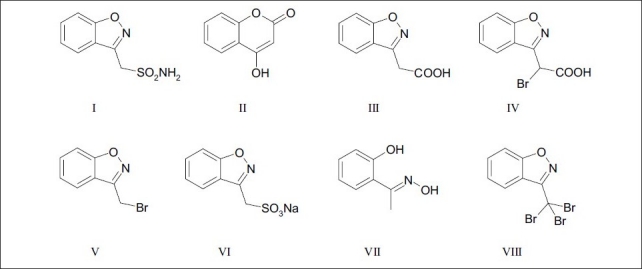
Structures of I-VIII I: 1,2-benzisoxazole-3-methanesulfonamide, II: 4-hydroxycoumarin, III: 1,2-benzisoxazole-3-acetic acid, IV: 1,2-benzisoxazole-3-bromoacetic acid, V: 1,2-benzisoxazole-3-methylbromide, VI: Sodium 1,2-benzisoxazole-3-methanesulfonate, VII: 2-hydroxyacetophenone oxime, VIII: 3,3,3-tribromomethyl-1,2-benzisoxazole.

## MATERIALS AND METHODS

The drug (I) and the impurities II-VIII were synthesized and characterized in-house. Potassium dihydrogen phosphate (AR grade), methanol (HPLC grade), acetonitrile (HPLC grade), orthophosphoric acid, hydrochloric acid, sodium hydroxide and hydrogen peroxide were procured from Merck India (Mumbai, India). The chromatographic separation was carried out on a Merck Hitachi HPLC system consisting of L-7100 pump, L-7300 Column oven, L-7200 Autosampler, L-7420 detector and D-7000 system manager data acquisition software EZ chrom elite version 3.1.2 (Merck KGaA, Darmstadt, Germany).

### Chromatographic conditions:

The mobile phase had two components; component A consisted of a mixture of 0.02 M aqueous potassium dihydrogen phosphate-acetonitrile-methanol (75:10:15 v/v/v), pH adjusted to 4.0 with orthophosphoric acid and component B consisted of a mixture of 0.02 M aqueous potassium dihydrogen phosphate-acetonitrile-methanol (15:40:45 v/v/v), wherein pH was adjusted to 2.0 with orthophosphoric acid. Both components A and B were filtered separately through a 0.45 μ nylon membrane and degassed under sonication before use. The chromatographic column used was Waters Symmetry C18 (150×3.9 mm, 5 μ, Waters Corporation, Milford, USA). The chromatography was performed at a flow rate of 1.0 ml/min using a linear gradient changing the component A (100%) to component B (100%) over a period of 45 min. The detection wavelength was 285 nm and injection volume was 20 μl.

### Method validation:

Stock solutions of 2 mg/ml of I and 0.002 mg/ml each of the impurities II-VIII were prepared in water. Additionally, a solution containing a mixture of 2 mg/ml of I and 0.002 mg/ml each of II-VIII was prepared for system suitability.

### Specificity:

A solution containing 2 mg/ml of I and 0.002 mg/ml each of II-VIII was analyzed on HPLC using the above-given method. Further, individual solutions of 2 mg/ml of I and 0.002 mg/ml each of II-VIII were analyzed to identify the individual peaks in the mixture.

### Limit of Detection (LOD) / Limit of Quantitation (LOQ):

A series of solutions containing I and the impurities II-VIII in the range of 0.001% to 0.01% were prepared for determining LOD and analyzed by using the above HPLC method. LODs were determined from visual observation of areas of I-VIII at each concentration in comparison with background obtained by injecting a blank. LOQs were considered as 3 times of LODs. Six injections each of solutions containing concentrations equivalent to LODs and LOQs of I-VIII were performed to establish precision.

### Linearity and Range:

The linearity of detector response of I-VIII at different concentrations was assessed covering an approximate range of 0.0035% to 0.2% (LOQ level to 200% of limit concentration) at six different concentration levels. Graphs of peak area against concentration were plotted for I-VIII using a linear regression model.

### Accuracy:

The accuracy was established by recovery studies by spiking 0.05%, 0.1% and 0.15% (with respect to sample concentration of 2 mg/ml) with previously prepared stock solutions of II-VIII. The specified limit for each impurity was not more than 0.10%. The analysis for each level was carried out in triplicate.

### Precision:

The precision of the method was established by the study of repeatability (system precision), reproducibility (method precision) and intermediate precision. The repeatability was checked by making six injections of a solution containing 1 mg/ml each of I-VIII and % RSD was calculated for peak areas obtained for I-VIII. For reproducibility, 2 mg/ml each of six different solutions of I were prepared and analyzed. Intermediate precision was performed by a second analyst on a different day using a different instrument.

### Robustness:

The robustness of the method was established by minor changes in chromatographic conditions by varying flow rate and pH of the mobile phase component A. The flow rates were changed from 1 ml/min to 0.9 ml/min and 1.1 ml/min, while pH was changed from 4.0 to 3.9 and 4.1. In all these experiments, concentration was 1 mg/ml each for the drug and II-VIII.

### Forced degradation studies:

Sample solutions of I were prepared at a concentration of 2 mg/ml each in 2N HCl, 2N NaOH and 10% H_2_O_2_ and were kept at room temperature. The drug was also exposed in solid and solution (2 mg/ml solution in water) to sunlight and UV rays. Solid samples of I were also kept at 105° and at melting point temperature of 158°. Additionally, 0.2% w/v solution of drug was prepared in water as a control sample. All the samples were analyzed at 0 hours and after 5 days by HPLC.

## RESULTS AND DISCUSSION

**Fig. 2:**
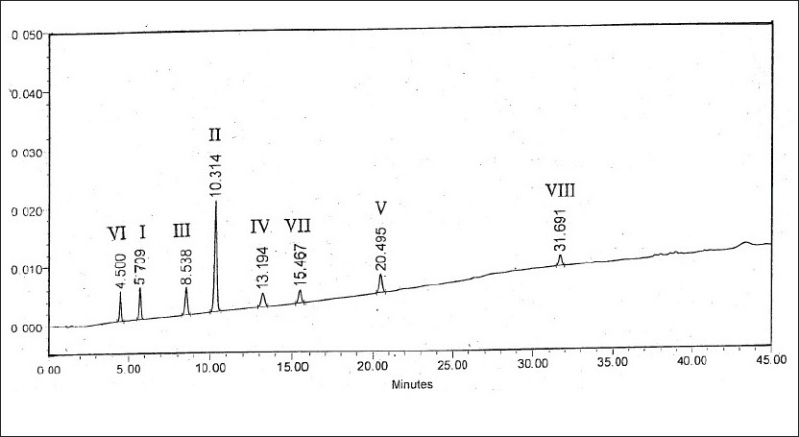
Chromatogram of a resolution mixture of I-VIII I: 1,2-benzisoxazole-3-methanesulfonamide, II: 4-hydroxycoumarin, III: 1,2-benzisoxazole-3-acetic acid, IV: 1,2-benzisoxazole-3- bromoacetic acid, V: 1,2-benzisoxazole-3-methylbromide, VI: Sodium 1,2-benzisoxazole-3-methanesulfonate, VII: 2-hydroxyacetophenone oxime, VIII: 3,3,3-tribromomethyl-1,2-benzisoxazole.

The objective was to develop a single HPLC method for the simultaneous detection and quantitation of all the known and unknown impurities in zonisamide (I). As per the reported route of synthesis[4], the known related substances in zonisamide (I) are the starting material II, intermediates III-VI and process impurities VII-VIII. Based on the absorption maxima observed for I-VIII, the detection wavelength was set at 280 nm. Different octadecylsilyl silica columns like Lichrospher RP 18e, Waters Symmetry, Hypersil ODS, and mobile phases comprising of phosphate, acetate and/or formate buffers in combination with methanol or acetonitrile or both were tried under gradient and isocratic conditions to develop a suitable HPLC method for the detection and quantitative determination of II-VIII in zonisamide (I). The effects of pH of mobile phase and column oven temperature on resolution between the components and tailing factors were also studied. Both column temperature and pH of the mobile phase were found to have a strong influence on the resolution and peak shapes. A reasonably good separation between the components with good peak shapes was achieved on a Waters Symmetry C8 (150×3.9 mm), 5 μ column using a linear gradient of mobile phase consisting of a mixture of 0.02 M potassium dihydrogen phosphate (anhydrous)-acetonitrile-methanol, pH 4.0 at a flow rate of 1.0 ml/min and column temperature at 35°. The linear gradient was 100% mobile phase component A (0.02 M aqueous potassium dihydrogen phosphate-acetonitrile-methanol (75:10:15 v/v/v), pH adjusted to 4.0 with orthophosphoric acid) to 100% mobile phase component B (0.02 M aqueous potassium dihydrogen phosphate-acetonitrile-methanol (15:40:45 v/v/v), pH 2.0 with orthophosphoric acid) over a period of 45 min. The retention times (RT) and relative retention times (RRT) obtained under these chromatographic conditions are presented in Table 1. The results indicated good resolution between the components with satisfactory peak shapes. A representative chromatogram depicting resolution between all the components I-VIII is shown in fig. 2.

**TABLE 1:** SPECIFICITY DATA FOR I-VIII

Component	RT (min)	RRT	Resolution	Asymmetry
I	5.7	1.00	6.4	1.0
II	10.3	1.81	6.9	0.9
III	8.5	1.50	12.5	0.9
IV	13.2	2.31	9.1	1.0
V	20.5	3.59	15.7	0.9
VI	4.5	0.79	-	1.0
VII	15.5	2.71	6.6	0.9
VIII	31.7	5.60	34.1	1.0

RT = Retention time; RRT = Relative retention time.

**Fig. 3:**
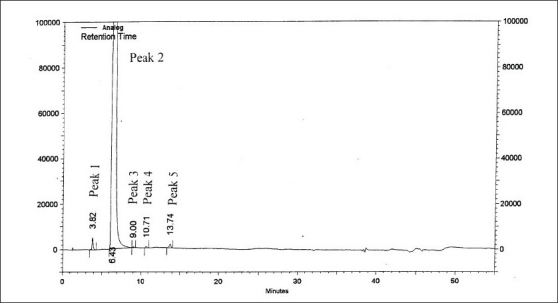
Chromatogram of a zonisamide sample Peak 1 is the unknown impurity, t_R_: 3.82 min, peak 2 is zonisamide, t_R_: 3.82 min and peaks 3-5 are also unknown impurities, t_R_: 9.00, 10.71 and 13.74 min.

Table 2 summarizes the results obtained for LOD, LOQ and linearity for compounds I-VIII. The LOD and LOQ values obtained for I-VIII were in the range of 0.001% to 0.007% and 0.0035% to 0.25% respectively (with respect to sample concentration of 2 mg/ml). The LOD and LOQ values were much below the specified limits (< 0.10%) for I-VIII, indicating that the method was sensitive and capable to detect and quantify known impurities II-VIII and also unknown impurities (fig. 3). Linearity was established in the range of LOQ level to 0.2% (limit concentration for II-VIII: < 0.10%) having regression coefficients in the range of 0.9958 to 0.9999.

**TABLE 2:** LOD, LOQ AND LINEARITY DATA FOR I-VIII^A^

Component	% LOD (% RSD)	% LOQ (% RSD)	Linearity (r^2^)
I	0.0035 (4.07)	0.01 (2.05)	0.01 % to 0.20 % (0.9958)
II	0.001 (2.83)	0.0035 (3.43)	0.0035 % to 0.20 % (0.9994)
III	0.003 (3.16)	0.01 (3.48)	0.01 % to 0.20 % (0.9999)
IV	0.0045 (2.58)	0.015 (2.28)	0.015 % to 0.20 % (0.9996)
V	0.004 (4.45)	0.012 (2.10)	0.015 % to 0.20 % (0.9980)
VI	0.005 (2.95)	0.015 (3.33)	0.015 % to 0.20 % (0.9998)
VII	0.005 (6.86)	0.015 (3.54)	0.015 % to 0.20 % (0.9999)
VIII	0.007 (4.84)	0.025 (2.50)	0.025 % to 0.20 % (0.9998)

a LOD, LOQ and Linearity values are in % with respect to sample concentration of 2 mg/ml.

The mean recoveries at each level for all the components I-VIII, given in Table 3, ranged between 97.2-100.7% (Acceptance criteria: 90-110%) and % RSD was between 0.1% and 2.7% (Acceptance criteria: NMT 10.0%) establishing that the method was accurate for the quantitative determination of all the known and unknown impurities.

**TABLE 3:** RECOVERY DATA FOR II-VIII^A^

Component	% Mean Recovery (% RSD)		
	
	Amount spiked = 0.05 %	Amount spiked = 0.10 %	Amount spiked = 0.15 %
II	99.81 (0.72)	98.56 (0.72)	99.73 (0.59)
III	98.01 (1.86)	99.48 (0.20)	98.79 (1.73)
IV	99.90 (0.53)	99.50 (0.10)	99.21 (0.48)
V	99.19 (0.14)	100.70 (2.68)	101.11 (0.93)
VI	99.79 (0.49)	99.64 (0.56)	100.71 (0.48)
VII	97.22 (1.80)	99.54 (0.83)	97.67 (1.07)
VIII	98.92 (0.38)	100.35 (0.58)	98.40 (1.05)

a % Amount spiked is with respect to sample concentration of 2 mg/ml.

The % RSD of peak areas for I-VIII were 1.46, 0.84, 3.08, 1.43, 1.19, 1.16, 1.40 and 1.51 respectively (acceptance limits: NMT 5.0%). This confirmed the repeatability of the method. In case of reproducibility, the contents of II-VIII and the unknown impurities (at RRTs 0.59, 1.48, 1.69 and 2.14) in all the six injections were found to be almost same and below 0.05%, thus establishing the reproducibility of the method. Intermediate precision, performed by second analyst on a different day using a different instrument gave results comparable with those of first analyst. The robustness results are summarized in Table 4. The resolution between I and VI was decreased, except in case of pH 4.1, while asymmetry was found to increase when the flow rate and pH of the mobile phase component A were changed. However, the chromatographic elution pattern remained unaffected, establishing the robustness of the method.

**TABLE 4:** EFFECT OF FLOW RATE AND pH CHANGES ON RESOLUTION AND SYMMETRY FACTOR

Parameter	Component	Method*	Flow rate (ml/min)		pH	
				
			0.9	1.1	3.9	4.1
Resolution (between I and VI)	-	6.40	5.35	5.43	6.01	6.88
Symmetry Factor	I	1.0	1.32	1.37	0.85	1.32
	II	0.9	1.28	1.29	1.22	1.25
	III	0.9	1.26	1.28	1.24	1.24
	IV	1.0	1.23	1.19	1.15	1.17
	V	0.9	1.29	1.21	1.27	1.27
	VI	1.0	1.38	1.41	1.39	1.40
	VII	0.9	1.29	1.24	1.31	1.18
	VIII	1.0	1.36	1.16	0.92	1.55

* Recommended method, wherein flow rate is 1 ml/min and pH of mobile phase is 4.0.

**Fig. 4:**
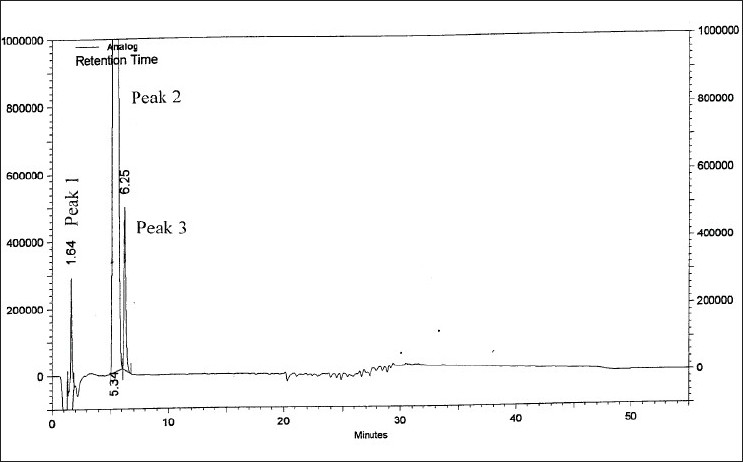
Chromatogram of zonisamide degraded under alkaline conditions Degradation under alkaline conditions was achieved by exposing to 2N NaOH/5 days at room temperature. Peak 1 is the degradation product, t_R_: 1.64 min, peak 2 is sodium 1,2-benzisoxazole-3-methane sulfonate as a degradation product, t_R_: 5.34 min and peak 3 is zonisamide, t_R_: 6.25 min.

**Fig. 5:**
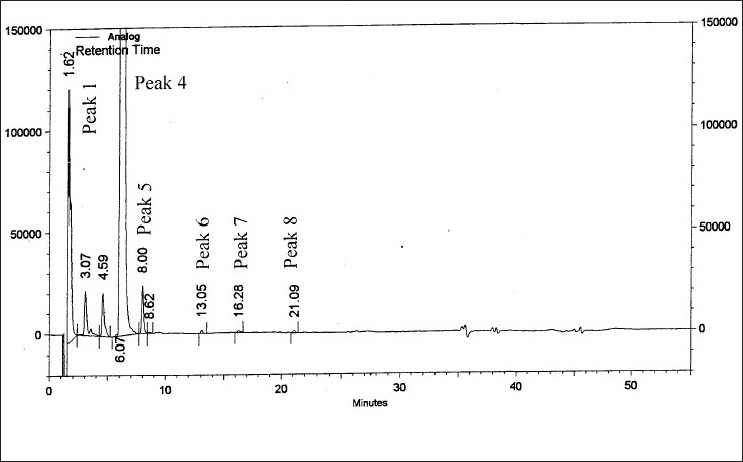
Chromatogram of zonisamide degraded under oxidative conditions Degradation under oxidative conditions, 10% H2O2/5 days at room temperature. Peaks 1-3 are degradation products, t_R_: 1.62, 3.07 and 4.59 min, Peak 4 is of zonisamide, t_R_: 6.07 min and peaks 5-8 are degradation products. t_R_: 8.00, 13.05, 16.28 and 21.09 min.

On forced degradation, no degradation was observed under acid, light and elevated temperatures, while under alkaline conditions, I was converted to VI by 91.89% by area normalization (fig. 4). Under oxidative conditions, I degraded by about 3.2%. All the degradation products (RRT 0.27:2.10%, RRT 0.50:0.44%, RRT 0.76:0.39% and RRT 1.32:0.33% by area normalization) were well resolved from the principal peak (fig. 5). The peak purity analysis of all the samples using PDA detector confirmed that there was no co-eluting peak in the principal peak of I, thus, establishing both impurity and stability-indicating nature of the method.

A single reversed-phase gradient HPLC method was developed and validated for the simultaneous detection and quantitation of known and unknown impurities formed during the synthesis of zonisamide (I). The method proved to be sensitive, selective, precise, linear, accurate and stability-indicating. The method was successfully applied to the analysis of I demonstrating acceptable precision and adequate sensitivity for the detection and quantitation of the impurities. So it may be reasonable to claim that the method can be extended to the analysis of drug formulations and stability samples as well.
